# Thrombospondin-1-N-Terminal Domain Induces a Phagocytic State and Thrombospondin-1-C-Terminal Domain Induces a Tolerizing Phenotype in Dendritic Cells

**DOI:** 10.1371/journal.pone.0006840

**Published:** 2009-08-31

**Authors:** Adi Tabib, Alon Krispin, Uriel Trahtemberg, Inna Verbovetski, Mario Lebendiker, Tsafi Danieli, Dror Mevorach

**Affiliations:** 1 The Laboratory for Cellular and Molecular Immunology, Rheumatology Research Unit, and The Protein Expression and Protein Purification Facilities, Hebrew University and Hadassah University Medical Center, Jerusalem, Israel; 2 Wolfson Centre for Applied Structural Biology, Hebrew University and Hadassah University Medical Center, Jerusalem, Israel; Charité-Universitätsmedizin Berlin, Germany

## Abstract

In our previous study, we have found that thrombospondin-1 (TSP-1) is synthesized *de novo* upon monocyte and neutrophil apoptosis, leading to a phagocytic and tolerizing phenotype of dendritic cells (DC), even prior to DC-apoptotic cell interaction. Interestingly, we were able to show that heparin binding domain (HBD), the N-terminal portion of TSP-1, was cleaved and secreted simultaneously in a caspase- and serine protease- dependent manner. In the current study we were interested to examine the role of HBD in the clearance of apoptotic cells, and whether the phagocytic and tolerizing state of DCs is mediated by the HBD itself, or whether the entire TSP-1 is needed. Therefore, we have cloned the human HBD, and compared its interactions with DC to those with TSP-1. Here we show that rHBD by itself is not directly responsible for immune paralysis and tolerizing phenotype of DCs, at least in the monomeric form, but has a significant role in rendering DCs phagocytic. Binding of TSP-1-C-terminal domain on the other hand induces a tolerizing phenotype in dendritic cells.

## Introduction

Mammalian thrombospondins are a group of matricellular proteins of complex spatial structure, which mediate a wide range of intercellular activities, and participate in cell-matrix interactions. This family includes five proteins, divided into two subfamilies, which possess different roles and tissue expression [1]. Group A, consisting of thrombospondins TSP-1 and TSP-2 in humans, also exists in invertebrates, and appeared prior to the development of the clotting and fibrinolytic systems of proteins, indicating their essentiality [2], [3].

TSP-1, the prototype thrombospondin, is a calcium-binding protein that plays an important role in the cellular response to various growth factors, cytokines, and inflammatory mediators [4], [5]. It controls proliferation, migration, and apoptosis in a variety of conditions, such as wound healing, inflammation, and neoplasia [2], through a large number of domains interacting with distinct receptors. However, there is a degree of overlap in its effects, which are mediated through different domains. Therefore, different or even opposing influences of the same protein may occur under certain circumstances, depending on the set of receptors expressed by the target cell [6].

In our previous study, we have found that TSP-1 is synthesized *de novo* upon monocyte and neutrophil apoptosis, leading to a phagocytic and tolerizing state of dendritic cells (DC), even prior to DC-apoptotic cell interaction [7]. Interestingly, we were able to show that heparin binding domain (HBD), the N-terminal portion of TSP-1, was cleaved and secreted simultaneously in a caspase- and serine protease-dependent manner.

In the current study we were interested to examine the role of HBD in the clearance of apoptotic cells, and whether the phagocytic and tolerizing state of DCs is mediated by the HBD itself, or whether the entire TSP-1 is needed. Therefore, we have cloned the human HBD, and compared its interactions with DC to those with TSP-1.

## Materials and Methods

### Media and Reagents

Dendritic cell culture medium consisted of RPMI 1640 with 1% L-glutamine, 1% penicillin/streptomycin (Biological Industries, Kibbutz Beit-Haemek, Israel), 1% autologous human plasma, and recombinant human cytokines GMCSF and IL-4 (R&D Systems, Minneapolis, MN or PeproTech, London, UK).

Ficoll-Paque was purchased from Amersham Pharmacia Biotech (Piscataway, NJ).

Mouse-anti-Human HLA-DR-FITC was obtained from Becton Dickinson (Franklin Lakes, NJ). Mouse-anti-human CD86-FITC and isotype controls were from Dako Cytomation A/S (Glostrup, Denmark). Green fluorescent latex beads (L-4655) and LPS were obtained from Sigma-Aldrich (St. Louis, MO). TSP-1 was obtained from Sigma-Aldrich and Protein Sciences (Meriden, CT). 1,1′-dioctadecyl-3,3,3′,3′-tetramethyl-indocarbocyanineperchlorate (DiI) was obtained from Molecular Probes (Eugene, OR).

Recombinant heparin binding domain [8] (rHBD2) was kindly provided by Prof. Jack Lawler, Department of Pathology, Harvard Medical School, Boston, MA.

### Protein Identification

SDS-PAGE. Gradient 4% to 20% polyacrylamide-SDS gels and SDS buffer were prepared according to the Laemmli method. The molecular mass of the protein bands was determined by means of a Precision Plus Protein Standards Kit (Bio-Rad Laboratories, Hercules, CA). Proteins were visualized using a silver-staining kit (Amersham Pharmacia Biotech) or Bio-Safe Coomassie (Bio-Rad), according to the manufacturer's instructions. Gel images were acquired using a Umax Power Look III scanner (Umax Systems, Willich, Germany).

Mass spectrometry of HBD and rHBD1 was performed using a Micromass Q-Tof system, equipped with a NanoFlow Probe Tip Type F (Micromass UK, Manchester, UK). The extracted peptide solution was collected in a borosilicate capillary tip (Protana, Odense, Denmark) and subjected to ESI at a flow rate of 10 nL/min. The MS spectra were analyzed with MicroMass Protein Lynx software. Protein identification was conducted using the MS-FIT proteomic tool from the Matrix-Science website (http://www.matrixscience.com). For rHBD1 identification and sequence verification, samples from selected FPLC fractions were separated using 12% SDS-PAGE gel. Proteins were then transferred to a nitro-cellulose membrane. The rHBD band was traced using polyclonal antibody directed to the N-terminal 20 amino-acid sequence of thrombospondin-1 (clone N-20, Santa-Cruz Biotechnology, Santa Cruz, CA) and donkey anti-goat IgG HRP (Promega, Madison, WI) followed by ECL reaction (Biological Industries).

### Cloning

Human throbospondin-1 N terminal c-DNA (Pubmed citation, accession No. NM_003246) containing HBD was purchased from RZPD (Berlin, Germany).

Primers, forward GGGAATTCCATATGAACCGCATTCCAGAGTCTGGCGG and reverse TGAATTATAAGCTTAGAGGACACTGGTAGAGCTGGAGC were purchased from Syntezza (Jerusalem, Israel). They were designed to exclude a 662 base-pair nucleotide sequence and included restriction sites to fit a pHis1 parallel bacterial vector. PCR product was then purified by gel extraction and a silica membrane containing column purification (Intron Biotechnology, Gyeonggi-do, Korea). Restriction reactions were preformed using Nde-I and Hind-III restriction enzymes (New England Biolabs, Ipswich, MA) followed by ligation reaction (Takara Bio, Shiga, Japan) to pHis parallel bacterial vector containing ampicillin resistance marker (Novagen, EMD Chemicals Inc. Darmstadt, Germany). Constructs of pHis1 parallel bacterial vector containing HBD inserts were transformed into TOP-10 competent E. coli (Kindly provided by Dr. P Sheffield from Dr. Z Derewenda, UVA, based on pET 22, Invitrogen, Carlsbad, CA).

Ampicillin containing Luria Broth (LB)-agar plates were used to isolate construct containing bacteria, from which a few were screened to identify insert containing plasmids using a mini-DNA purification kit (Intron Biotechnology), followed by diagnostic restriction reactions. Insert-containing constructs were then transformed to Origami B expressing bacteria. Origami B strain, transformed with pHis1 empty vector was used for control.

### Expression and Purification of rHBD

1.5 L of fresh LB medium containing ampicillin was inoculated with 50 ml of starter culture, incubated at 37°C to A600 = 0.6, and then at 22°C for 30 min. Protein expression was induced by the addition of 0.1 mM of isopropyl-β-D-thiogalactopyranoside (IPTG) to the culture media, and incubation overnight at 17°C.

Cells were collected by centrifugation and stored at −70°C. For lysis, cell pellets were thawed on ice and suspended in buffer A (0.1 M NaCl, 20 mM Tris–HCl buffer pH 8.0); supplemented with 10 mM MgCl_2_, PMSF (1 mM), lysozyme (0.2 mg/ml), and DNaseA (50 ug/ml); and disrupted mechanically using micro-fluidizer (model M-110 EHIS; Microfluidics Corp., Newton, MA). The soluble and insoluble phases were separated by centrifugation (20,000 g for 20 min at 4°C). Chromatography was performed using the AKTA Explorer FPLC system (Amersham Biosciences, Piscataway, NJ). Supernatant was loaded on a buffer A pre-equilibrated Q-Sepharose column (Amersham Pharmacia) in tandem with a buffer A pre-equilibrated heparin-agarose column (Amersham Pharmacia) and extensively washed. Proteins were then eluted from the heparin-agarose column using a step gradient of NaCl in buffer A. Protein fractions were pooled according to their molecular weight pattern of SDS-PAGE separation and concentrated using AMICON concentrator centrifugal devices with molecular weight cutoff of 10,000 kDa. For further purification, fractions were applied to a Sephacyl S100 column (96×2.6 cm) using an AKTA FPLC, and equilibrated with PBS buffer. The protein was quantified using spectrophotometry at 280 nm, supplemented with 20% glycerol, and then aliquoted and preserved at −80°C.

### Western Blotting

Samples from selected FPLC fractions were treated with sample-bufferX5, boiled at 95°C degrees for 5 minutes, and analyzed through SDS-PAGE 12% separating gel (1.5 Tris HCl pH 8.3, 40% acrylamide\bisacrylamide 1∶19 Sigma Aldrich). Proteins were then transferred to a nitro-cellulose membrane. rHBD was traced using polyclonal goat IgG α-HBD N-20 (Santa Cruz Biotechnology) and donkey anti-goat IgG HRP (Promega, Madison) followed by ECL reaction (Biological Industries).

### Generation of Monocyte-Derived Dendritic Cells

Immature monocyte-derived dendritic cells (iDCs) were generated from the CD14^+^ selected fraction of PBMCs and from blood donors' buffy coats. iDCs were isolated as described [9]. Briefly, PBMCs were isolated using Ficoll and anti-CD14 magnetic beads in order to isolate monocytes from PBMCs according to the manufacturer's instructions (Becton Dickinson). iDCs were placed in wells at a concentration of 1.25×10^6^/1.5 ml culture media in the presence of 1% autologous plasma, GMCSF, and IL-4. Every 2 days, 0.15 ml was removed and 0.25 ml of media containing plasma, IL-4, and GMCSF, 500 U/ml, was added. On day 6, iDCs were harvested, washed, and counted.

### Induction and Detection of Monocyte Apoptosis

Monocytes were obtained from buffy coats, using CD14 magnetic beads (Beckton Dickinson) according to the manufacturer's instructions. Cells were then stained with 5 µg/ml DiI in RPMI and incubated on ice for 30 minutes. Serum withdrawal apoptosis was used for the generation of apoptotic monocytes in serum-free RPMI. Monocytes were plated at a concentration of 6.6×10^6^/ml in 35 mm diameter dishes for 12 hour incubation at 37°C. Apoptosis was detected using Annexin V and propidium iodide (PI) staining by flow cytometry as previously described [7]. Confirmation of apoptosis was made by hypodiploid PI staining and loss of mithochondrial membrane potential.

### Interaction of Apoptotic Cells with iDCs

Interaction was performed as we have described [7]. Briefly, after harvesting iDCs, cells were replated in 96-well plate (Corning, New York, NY) at a concentration of 2.5×10^5^ in 300 µl iDC culture medium. Serum withdrawal, DiI-labeled apoptotic cells were offered to iDCs at a 1∶4 iDCs:apoptotic monocyte ratio for four hours of interaction. iDC uptake of apoptotic monocytes was read using FACScan™ (Becton Dickinson). iDC uptake of DiI-stained apoptotic monocytes was evaluated by mean DiI staining of DC-SIGN-FITC positive DCs. For DC maturation assays, unlabeled apoptotic monocytes were offered to iDCs as described above, and after four hours of co-incubation, LPS (10 µg\ml, Sigma-Aldrich) was added. The expression of DR and CD86 was evaluated 20 hours later, using flow cytometry.

### Inhibition Assays

iDCs were exposed to several anti-HBD blocking antibodies at day 6 as follows: anti-HBD (N-20, Santa Cruz Biotechnology), anti-β1 integrin (CD29, Chemicon, Boronia Victoria, Australia), and anti-CD91 alpha- and beta chain (American Diagnostica, Stamford, CT). Cells were then washed, and apoptotic monocytes stained with DiI were offered in the presence or absence of HBD. iDC acquisition of DiI was measured by flow cytometry. iDCs were separated from monocytes based on DC-SIGN-FITC staining.

### rHBD Binding Assays

iDCs were washed with RPMI and incubated for 15 minutes on ice with 10 µg\ml rHBD. Cells were then washed and incubated with anti-HBD (N-20, Santa Cruz Biotechnology), followed by secondary antibody, FITC-conjugated donkey anti-goat (Santa Cruz Biotechnology). Binding was evaluated using flow cytometry.

### Statistics

Statistical comparisons of mean data were performed using one-way analysis of variance (ANOVA) and the Students t-test with Bonferroni correction for multiple comparisons. The Students t-test was also used to compare uptake, and to compare the expression of surface molecules on DCs.

## Results

### Generation of Recombinant Heparin Binding Domain of Human Thrombospondin-1

cDNA containing the desirable sequence was amplified using polymerase chain reaction. The reaction utilizes a 662 base pair sequence that encodes 221 amino-acids of HBD (residues 19–240), including Cys-150 and Cys-214, which participate in intrachain disulfide bond formation, and lack the leader peptide (residues 1–18). PCR fragments were tested both through 1% agarose gel, in which we had a clear solitary band representing the PCR product located between 700 and 500 DNA 100 bp ladder, and sequencing (The center for genomic technologies, Hebrew University, Jerusalem).

pHis1 parallel bacterial vector ligated with HBD inserts and transformed into Origami B-expressing bacteria strain. As control, we used Origami B transformed with pHis1 empty vector.

Transformed bacteria were incubated in auto-induced media (5052-ZYP) for primary investigation of protein expression. Coomassie staining showed protein presence in Origami B supernatant after sonication, but not in the control system. Protein expression induction was performed overnight at 17°C with 100 µg\ml IPTG.

Protein was then purified using a heparin agarose bead column followed by further purification via gel filtration.

**Figure 1:**
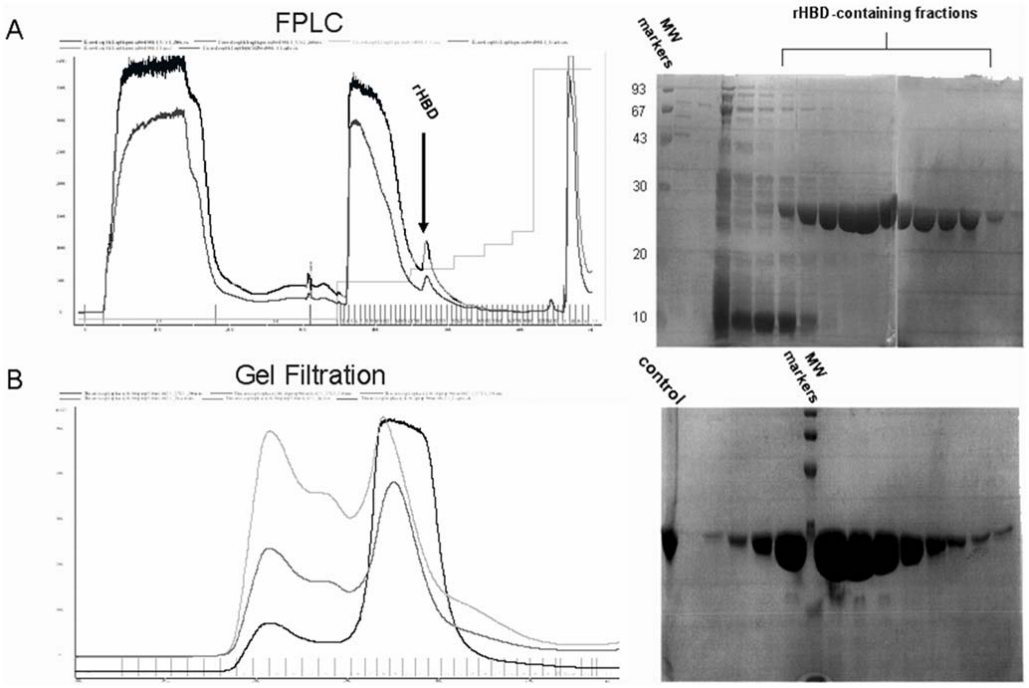
Cloning, expression and purification of the heparin binding domain (HBD) of thrombospondin-1. A. Fast protein liquid chromatography (FPLC) of heparin-agarose purified rHBD. HBD constructs were transformed into competent E. coli, amplified, verified, and extracted for expression in expressing bacteria (Origami B), as described in Materials and Methods. Bacteria were lysed, and the rHBD was separated from the supernatant using FPLC equipped with a heparin-agarose column. The rHBD location was approximated using the FPLC fraction flow curve, as shown (left). The black line represents protein content of fractions eluted from the column. The peak containing the rHBD is marked. The dark gray line probably represents DNA remnants. SDS-PAGE served for rHBD identification. Clear, growing bands, located between 20–30 kDa, represent eluted rHBD. MW = molecular weight. Light gray line represents gradient B (elution salt gradient). B. Gel filtration. In order to obtain highly purified rHBD, FPLC protein-containing fractions were combined and separated using a molecular weight exclusion column. Protein content was determined using fraction-curve and SDS-PAGE. Black line = rHBD elution curve; Dark and light gray lines represent nonprotein side products. SDS-PAGE indicates the presence of a substantial amount of protein, located between 20–30 kDa. Control = rHBD; verified by mass spectrometry

Recombinant HBD was further purified through gel filtration. The rHBD1 was verified through Mass spectrometry (The core research facility, Hebrew University, Jerusalem, Israel). Protein was than aliquoted and preserved at −80°C with 20% glycerol, and SDS-PAGE analysis (Fig. 1). Protein was then aliquoted and preserved at −80°C with 20% glycerol.

### Isolated Recombinant Heparin Binding Domain of Thrombospondin-1 Enhances Apoptotic Monocyte Engulfment

**Figure 2:**
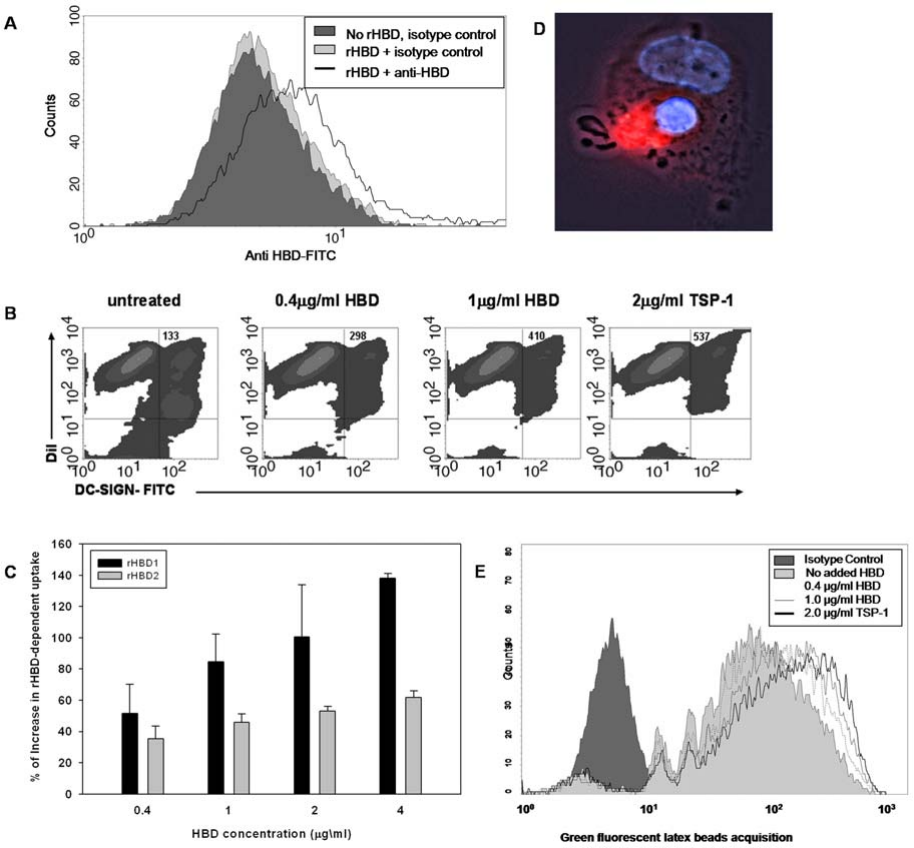
The isolated heparin binding domain of thrombospondin-1 enhances the specific- and nonspecific engulfment capacity of iDCs. A. rHBD binds immature dendritic cells. iDCs were washed twice with RPMI, and were either incubated for 15 min on ice with 10 µg/ml rHBD or left untreated. Cells were then rewashed and stained with anti-HBD antibody (N20, Santa Cruz Biotechnology, Lot. F0804), fluorescence-labeled secondary antibody, as described in Materials and Methods, or fluorescence-labeled isotype control (p<0.00034). B and C. rHBD enhances apoptotic monocyte engulfment by iDCs. Apoptotic monocytes were stained with 5 µg/ml DiI and offered to iDCs, at a 4∶1 ratio. A control group of iDCs were not exposed to apoptotic monocytes. Acquisition of apoptotic cell-derived DiI by the DCs was measured after 8 h of interaction. iDCs were separated from monocytes based on DC-SIGN-FITC staining. Results are representative of three experiments. B. Comparison between the engulfment effect of rHBD and TSP-1 on DiI-stained apoptotic monocytes. Dot plot of DC-SIGN positive cells (iDCs) and DiI-stained apoptotic monocytes. Mean fluorescence of DiI acquisition is indicated in the right upper quadrant. Pre-incubation of the iDCs with 0.4 µg/ml HBD significantly enhanced apoptotic monocyte acquisition by iDCs; median fluorescence was 298 compared to 133 for iDCs exposed to apoptotic monocytes without addition of rHBD. HBD, at 1.0 µg/ml, further enhanced iDC engulfment capacity with median fluorescence 410. The effect of TSP-1, 2.0 µg/ml, is indicated for comparison, with median fluorescence 537. Results are representative of three experiments. See also Figure 2C. C. Comparison between the effects of rHBD from different sources (rHBD1, black bars; rHBD2, grey bars) on iDC uptake of apoptotic monocytes. Y axis represents the percentage increase in rHBD-dependent uptake of apoptotic monocytes by iDCs as measured by DiI fluorescence, compared with baseline uptake when no rHBD is added. A dose-dependent augmentation of uptake is seen with the addition of rHBD from both sources. Results are mean±SE of three experiments. D. Human monocyte-derived dendritic cell engulfing an apoptotic monocyte. Wide field fluorescence microscopy image of a human monocyte-derived dendritic cell that has engulfed a DiI-stained apoptotic monocyte (red). DAPI (blue) was used to demonstrate the nuclei, with an overlay of the phase contrast profile. Note the difference between the lightly fluorescent dendritic cell nucleus and the condensed and highly fluorescent chromatin of the apoptotic monocyte nucleus. Original magnification, 1000×. E. Isolated HBD enhances latex bead engulfment by iDCs. Green fluorescent latex beads were offered to iDCs at a 16∶1 ratio, in the presence or absence of 0–1 µg/ml isolated HBD or 2 µg/ml TSP-1. Median fluorescence was enhanced from 55.67 (average of two measurements) with no added HBD, to 81.33 in the presence of 0.4 µg/ml HBD, and 90.14 in the presence of 1 µg/ml HBD, indicating augmentation in phagocytic capacity of 46.08% and 61.9% respectively, following HBD exposure (p<0.001). This is compared with 98.38% augmentation of phagocytosis in the presence of 2 µg/ml TSP-1. Data is representative of three experiments.

To test the hypothesis that HBD interaction with iDCs leads to a phagocytic state, expressed by enhanced engulfment ability, we first added HBD to iDCs in order to verify binding. As shown in Figure 2A, compared to isotype controls (mean fluorescence 4.8), rHBD binds iDCs (mean fluorescence 6.7, p<0.0003).

As we used TSP-1 concentrations similar to levels that were found secreted upon monocyte apoptosis [7], we used equimolar concentrations of rHBD in order to compare the effects of the rHBD to those of TSP-1. As shown in Figure 2B and C, this resulted in a dose-dependent increase in iDC engulfment of DiI-stained apoptotic cells. As shown in Figure 2C, mean iDC apoptotic cell acquisition was elevated in 51 and 84 percent when 0.4 and 1 µg\ml of HBD was added, respectively (three experiments, p<0001). Mean apoptotic cell acquisition upon addition of 4 µg\ml rHBD, (138 percent increase) was close to the effect of TSP-1 (162 percent increase). iDC engulfment of apoptotic monocytes was confirmed by fluorescent microscopy (Figure 2D).

To verify whether HBD of different origins would have a similar effect, we used rHBD from an alternative source (rHBD2, kindly provided by Jack Lawler, Harvard Medical School, Boston, MA). Comparison of the rHBDs suggested that both had a significant effect, although rHBD that we generated (rHBD1) was slightly more effective (Fig. 2C).

Finally, we wanted to see whether this pro-phagocytic effect is expressed when other, nonspecific particles are offered, and we offered green fluorescent beads to iDCs. As shown in Figure 2E, green fluorescent bead uptake was augmented when iDCs were exposed to rHBD.

### CD29, CD91, or N20 Blockade Abrogates the HBD-Dependent Engulfment Effect

HBD possesses several identified receptors. In complexes with calreticulin (CRT), CD91 was shown to be involved in apoptotic cells clearance [10], [11]. CD29 (β1 integrin) is known as an HBD receptor that mediates neurite growth and adhesion of cells in association with integrins α3, α4, or α5 (reviewed by Chen, et al. [12]). This receptor has not been clearly shown to be involved in apoptotic cell engulfment.

**Figure 3:**
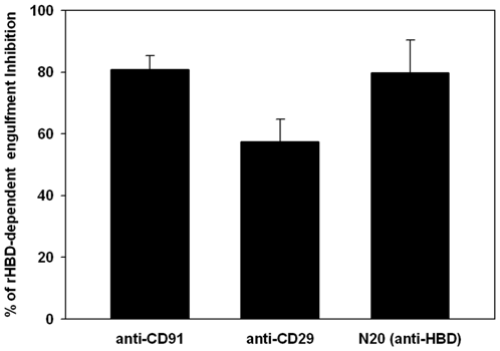
HBD-dependent apoptotic monocyte engulfment may be mediated through different receptors. Immature DCs were pre-incubated with antibodies against CD29 or CD91, and were then offered apoptotic monocytes in the presence of 0.4 µg/ml recombinant HBD. HBD was also added to dendritic cells in the presence of an antibody raised against the HBD (N20). Striking (≥90%) inhibition of HBD-dependent apoptotic monocyte uptake is seen upon blocking HBD binding to either CD29 or CD91, compared with almost complete blocking of the HBD effect with the addition of N20. The effect of each blocking antibody on rHBD-dependent engulfment is measured relative to the difference in engulfment between additions of isotype control with 2 µg/ml HBD to that without HBD. Data is a mean±SE of three experiments.

We conducted inhibition assays with antibodies specifically directed against each of these three receptors to examine whether they may mediate the HBD-dependent enhanced engulfment capacity of DCs. Surprisingly, both monoclonal specific antibodies resulted in a blockage of HBD influence, similar to that of the N-20, an antibody directed against HBD (Fig. 3).

### The Heparin Binding Domain of TSP-1, in Contrast to the Intact TSP-1, Does Not Induce Immune Paralysis

To test the hypothesis that the isolated HBD could inhibit iDC maturation as has been shown for TSP-1 [7], we incubated iDCs with either 0.4 µg/ml rHBD, an equimolar concentration to that of the TSP-1 secreted by apoptotic monocytes at 10 hours of apoptosis, or with 2.0 µg/ml TSP-1, and examined the changes in the iDC morphology and maturation profile using flow cytometry.

**Figure 4:**
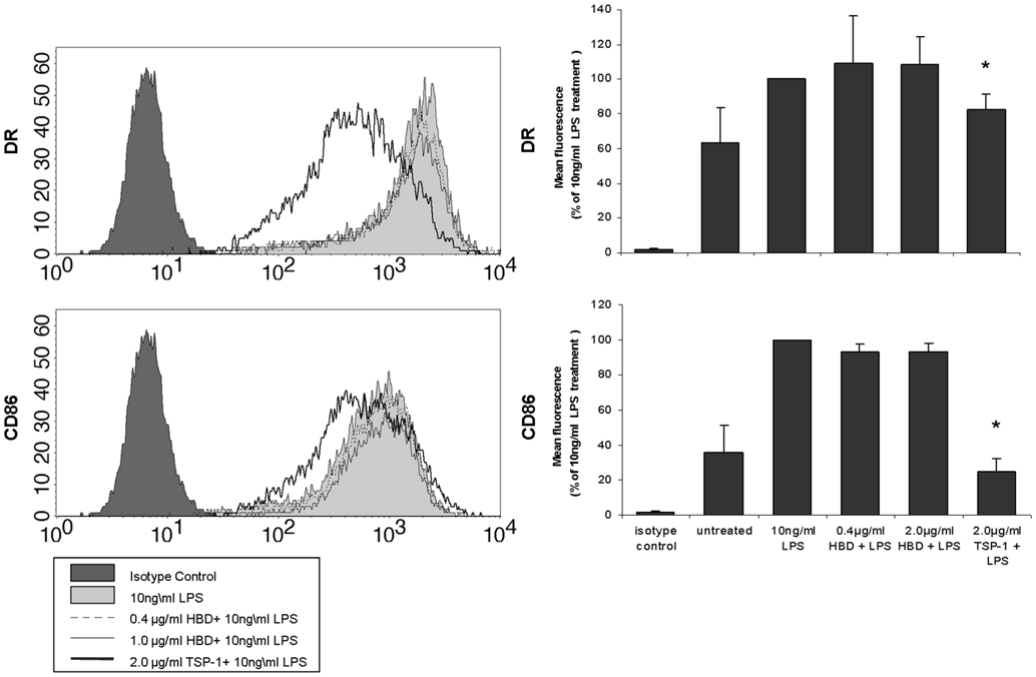
Effect of rHBD and TSP-1 on LPS-stimulated iDCs. A. Pre-incubation with HBD does not inhibit the expression of maturation-related molecules DR (upper chart) and CD86 (lower chart) when iDCs are exposed to LPS, whereas pre-incubation with TSP-1 at equimolar concentration inhibits their expression. Data is representative of three experiments. B. Compared to treatment with 10 ng/ml LPS, pre-incubation with different concentrations of HBD (0.4 or 1.0 µg/ml) before exposure to LPS did not have significant influence on expression of either DR (upper chart) or CD86 (lower chart). TSP-1, on the other hand, resulted in a decreased expression of both molecules, similar to that of untreated iDCs. Results are expressed as a percent of the expression of each molecule at the 10 ng/ml LPS treatment, and represent mean±SE of three experiments. Statistically significant difference is marked (*).

Surprisingly, the expression of maturation-related molecules MHC class II and CD86 was not inhibited by pre-incubation with isolated rHBD, compared to the effect of TSP-1 (Fig. 4), for a wide range of rHBD concentrations (Fig. 4B). In support of this finding, we further found that pre-incubation with rHBD did not influence the forward- and side-scatter features of DCs, whether exposed or not exposed to LPS. Addition of the whole TSP-1 resulted in a decrease in cell size along with increased granularity (data not shown).

## Discussion

Thrombospondin-1 controls proliferation, migration, and apoptosis in a variety of conditions, including wound healing and inflammation. The most important receptors identified to date for the varying effects moderated by TSP-1 are CD91, syndecan-1, and alpha3beta1 (α3β1) integrin for HBD; CD47 for the C terminal domain, CD36 HSPG and beta1 (β1) integrins for type I repeats; and alpha v beta 3 (αvβ3) for type III repeats. Different proteins in vertebrates and invertebrates include domains with similar structure and function to those of HBD, as reviewed in Adams and Lawler [2].

We showed earlier that TSP-1 induced a phagocytic and tolerizing state for iDCs [7]. In this study we were interested to examine the specific role that the isolated rHBD may play in this interaction. Our interest is due not only to the importance of understanding TSP-1 functions, but also because HBD is discretely secreted from apoptotic monocytes [7] and platelets [13]. For this purpose we have cloned and expressed recombinant monomeric HBD (rHBD) using human throbospondin-1 N-terminal domain c-DNA.

HBD, as part of TSP-1, was suggested to bind multiple receptors, including syndecan-1, syndecan-4, perlecan, Decorin, CD91 and its complex with calreticulin, VLDL-receptor, and integrins α6β1, α4β1, andα 3β1, as reviewd by Elzie et al. [13]. Here we were able to show that rHBD by itself can make iDCs better phagocytes of apoptotic cells. What could be the underlying mechanism? According to the model suggested by Savill and colleagues [14], TSP-1 may serve as a bridging molecule in the engulfment of apoptotic cells. We have shown that TSP-1 alone, without the presence of apoptotic cells, makes iDCs more phagocytic [7], Thus, it is likely that this HBD effect is achieved via signaling events following its binding. Using monoclonal antibodies, we were able to show that this effect was abrogated by inhibiting the action of CD29 and CD91, which are most likely involved in the signaling cascade. CD29, an integrin family member, is a membrane receptor involved in cell adhesion and recognition for a variety of processes, including embryogenesis, hemostasis, tissue repair, and immune response. CD29 was not previously known to be involved in phagocytosis. Here we show for the first time that CD29 may be required for HBD-dependent engulfment activity.

Interestingly, and in contrast to the effect of HBD as part of TSP-1, no inhibition of DC maturation by the rHBD was documented in our system. In other words, whereas isolated HBD mediates the phagocytic state it does not mediate the tolerizing phenotype of DCs. This can be due to several reasons. The first is that HBD is not required for DC maturation inhibition. In a previous work performed by our group [7] we showed that blocking antibodies directed against TSP-1 C-terminus and type 1 repeats (CD47, CD51 and CD36 respectively) inhibited up to 80% of the TSP-1 tolerizing phenotype. Thus, the tolerance effect could very well be mediated by interaction of the TSP-1 C terminus with CD47 [15], [16], or interaction of the type I repeats with CD36 [17]. Another possibility is that HBD plays a role in creating the tolerizing phenotype, but only as a part of TSP-1, when a signalosome is formed [7]. Indeed, some actions mediated by TSP-I require simultaneous interactions in other regions of the molecule [18] where intracellular crosstalks, mediated simultaneously through several receptors, are necessary. Our finding that blockage with anti-HBD (N20, Santa Cruz) did inhibit development of a tolerizing phenotype [7] supports this concept. An additional explanation may be a two-step mechanism, for example augmentation of apoptotic cell uptake that leads later to a more pronounced tolerizing phenotype. Thus, HBD is essentially a prophagocytic protein that enhances phagocytosis of apoptotic cells when binding to an iDC. Binding of TSP-1 as well as Annexin-1, phosphatidylserine, and probably additional molecules, allows DC immune paralysis. Finally, it is possible that trimeric HBD is needed for the full HBD effect, as it has been shown that trimetric HBD may act differently from monomeric HBD [19], [20].

There was some difference in phagocytic efficiency in the two rHBD samples. Our rHBD lacks the leader peptide (the first 19 amino acids), which is present in the rHBD2. The rest of the protein is completely identical. Both rHBD samples significantly increased phagocytic state and did not alter maturation, however activity was somewhat reduced in the rHBD2 sample. It seems that the cause for this difference is storage conditions, which preserve the HBD's biological activity. Indeed, we have found that storage of rHBD1 in 20% glycerol at −80°C protects the best biological activity, whereas storage at −20°C in PBS, the storage regimen for the rHBD2, activity was reduced (not shown).

In summary, we have shown that rHBD by itself is not directly responsible for immune paralysis and tolerizing phenotype of DCs, at least in the monomeric form, but has a significant role in rendering DCs phagocytic. Indirectly, as a part of TSP-1, or in its trimeric form, rHBD may influence development of the tolerizing phenotype of DCs following binding of TSP-1.
